# APTES monolayer coverage on self-assembled magnetic nanospheres for controlled release of anticancer drug Nintedanib

**DOI:** 10.1038/s41598-021-84770-0

**Published:** 2021-03-11

**Authors:** V. C. Karade, A. Sharma, R. P. Dhavale, R. P. Dhavale, S. R. Shingte, P. S. Patil, J. H. Kim, D. R. T. Zahn, A. D. Chougale, G. Salvan, P. B. Patil

**Affiliations:** 1 School of Nanoscience and Technology, Shivaji University, Kolhapur, Maharashtra 416004 India; 2 Optoelectronic Convergence Research Center and Department of Materials Science and Engineering, Chonnam National University, Gwangju, 500-757 South Korea; 3 Semiconductor Physics, Chemnitz University of Technology, 09107 Chemnitz, Germany; 4 Department of Materials Science and Engineering, Yonsei University, Seoul, 03722 South Korea; 5 Department of Pharmaceutics, Bharati Vidyapeeth College of Pharmacy, Kolhapur, Maharashtra 416013 India; 6 Department of Physics, The New College, Shivaji University, Kolhapur, Maharashtra 416012 India; 7 Department of Physics, Shivaji University, Kolhapur, Maharashtra 416004 India; 8 Department of Chemistry, The New College, Shivaji University, Kolhapur, Maharashtra 416012 India

**Keywords:** Materials science, Biomaterials, Condensed-matter physics, Nanoscale materials

## Abstract

The use of an appropriate delivery system capable of protecting, translocating, and selectively releasing therapeutic moieties to desired sites can promote the efficacy of an active compound. In this work, we have developed a nanoformulation which preserves its magnetization to load a model anticancerous drug and to explore the controlled release of the drug in a cancerous environment. For the preparation of the nanoformulation, self-assembled magnetic nanospheres (MNS) made of superparamagnetic iron oxide nanoparticles were grafted with a monolayer of (3-aminopropyl)triethoxysilane (APTES). A direct functionalization strategy was used to avoid the loss of the MNS magnetization. The successful preparation of the nanoformulation was validated by structural, microstructural, and magnetic investigations. X-ray photoelectron spectroscopy (XPS) and Fourier transform infrared spectroscopy (FTIR) were used to establish the presence of APTES on the MNS surface. The amine content quantified by a ninhydrin assay revealed the monolayer coverage of APTES over MNS. The monolayer coverage of APTES reduced only negligibly the saturation magnetization from 77 emu/g (for MNS) to 74 emu/g (for MNS-APTES). Detailed investigations of the thermoremanent magnetization were carried out to assess the superparamagnetism in the MNS. To make the nanoformulation pH-responsive, the anticancerous drug Nintedanib (NTD) was conjugated with MNS-APTES through the acid liable imine bond. At pH 5.5, which mimics a cancerous environment, a controlled release of 85% in 48 h was observed. On the other hand, prolonged release of NTD was found at physiological conditions (*i.e.*, pH 7.4). In vitro cytotoxicity study showed dose-dependent activity of MNS-APTES-NTD for human lung cancer cells L-132. About 75% reduction in cellular viability for a 100 μg/mL concentration of nanoformulation was observed. The nanoformulation designed using MNS and monolayer coverage of APTES has potential in cancer therapy as well as in other nanobiological applications.

## Introduction

The contemporary treatments for cancer are usually based on invasive surgery, chemotherapy, radiotherapy, immunotherapy, or a combination of these methods^1–4^. Even though chemotherapy is the most common treatment in practice, the imprecision in chemotherapeutic drug delivery at the tumour site results in adverse side effects due to the internalization of drugs by healthy cells along with tumour cells^5,6^. Researchers around the globe have addressed this issue and have offered different strategies to accomplish site-specific drug release^7–9^. A targeted drug delivery (TDD) system is currently considered to be the best alternative for systemic therapy. TDD can significantly improve the potency of the therapeutic drug^10,11^. Recently numerous drug carriers have emerged with their potential applicability as nanovectors in cancer treatment^12–14^. These drug carriers are comprised of nanoscale and supramolecular drug delivery systems which includes purely organic free radicals, nano-antioxidants, hydrophilic-hydrophobic polymeric nanoparticles (NPs), micelles, liposomes and dendrimers^15–18^. Besides some inorganic multifunctional nanoformulations such as magnetic-layered double hydroxide, multicore-shell nanostructure, or disk-shaped magnetic NPs (MNPs), magnetic nano-cubes with a magneto-mechanical approach have also been explored^13,19,20^. Among these carriers, different magnetic nanostructures receive growing attention as a promising theranostic tool, as their unique superparamagnetic nature allows the translocation of anticancer drugs at a chosen site through an applied magnetic field while colloidal stability of carriers in the body fluid is maintained^21,22^. However, to use these magnetic nanostructures in a TDD system, they must hold several properties such as biocompatibility, high surface area, high colloidal stability, superparamagnetism, and high saturation magnetization (M_s_)^23^. In this work, we used self-assembled magnetic nanospheres (MNS) made of iron oxide NPs as drug carriers, which exhibit superparamagnetic nature with a decent magnetic response compared to other magnetic nano-formulations and to other magnetic nanostructures^24^.

For TDD applications, the surface modification of MNPs is essential to load the drug on the MNP surface, restrict the interaction of MNPs with the host cells, and diminish the interparticle interaction. Several surface modification strategies have been employed for the functionalization of MNP surfaces. Different organic and inorganic coatings such as chitosan^25^, fatty acids^25^, polyacrylic acid^26^, polyvinylpyrrolidone^27^, polyethylene glycol^28^, polyvinyl alcohol^29^ were used for the surface modification. However, most of them are high molecular weight biomolecules (polymers), and all these multilayered coatings cause a decrease in magnetization M_s_ after the functionalization process^30^. Landarani-Isfahani et al*.*^31^ reported a decreasing magnetization of MNPs with an increase in the thickness of the polymer coating. Reduced magnetization deteriorates the MNP functionality as a drug carrier due to the lower response to an external magnetic force. On the other hand, MNPs with higher magnetization can aid effective delivery. Huang et al*.*^32^ demonstrated the delivery of theranostic agents across the blood–brain barrier in rats using MNPs under an external magnetic field. They observed a significant accumulation of MNPs in the cortex near the magnet and higher magnetization of functionalized MNPs could facilitate the accumulation of MNPs at a target site.

Silane derivatives-based functionalization strategies possess several advantages over other linkers. They improve the structural stability of MNPs by hindering oxidative damage through inert silane linkage coating, besides modify hydrophilicity or hydrophobicity for drug conjugation with functional groups like amine, sulfonyl, and carboxylic moieties^33–36^. 3-aminopropyl triethoxysilane (APTES) is an amino-silane linker frequently used in surface modification of MNPs^37–40^. Several studies reported the use of nonmagnetic SiO_2_ coating over the MNP surface for APTES surface modification, which in turn reduces the magnetization^41,42^. In this regard, it is necessary to adapt the optimal functionalization strategy that does not affect the MNP magnetic properties. In this work, we demonstrate the direct functionalization (without SiO_2_ shell) and monolayer coverage of APTES as an effective approach to retain the magnetic properties of MNS. The formation of a monolayer of amino silane eases the linking of a specific drug through the tale side amino groups (–NH_2_). These amino groups form the imine bond (C=N) between the carbonyl group of drug and amine group of MNS-APTES and assist the drug conjugation process.

**Scheme 1:**
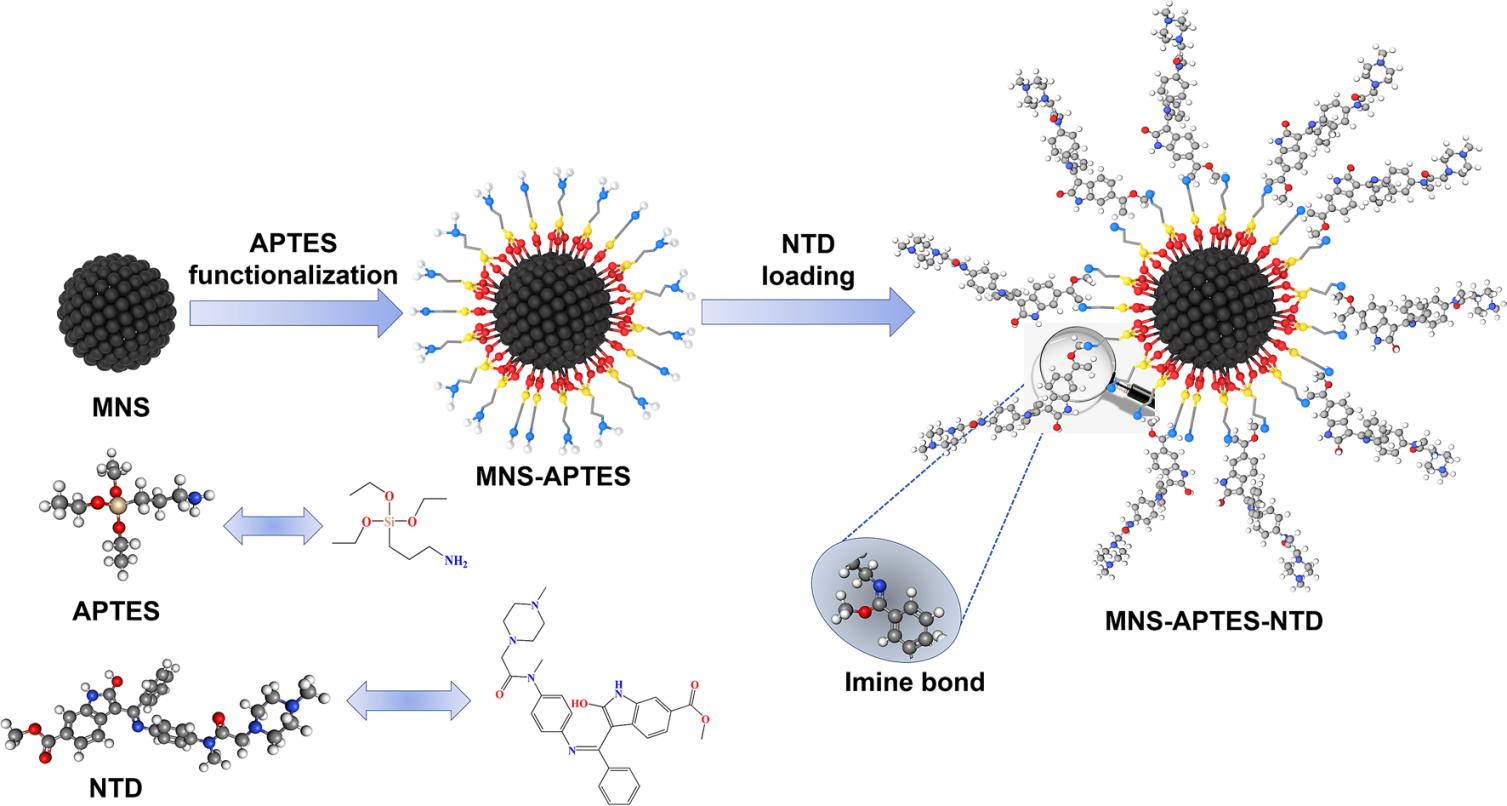
Schematic representation of the surface modification and loading of the drug over the MNS surface.

Nintedanib (NTD) is a receptor tyrosine kinase (RTK) inhibitor with antiangiogenic and antineoplastic potential which can target three angiogenesis related transmembrane receptors, namely the vascular endothelial growth factor receptor (VEGFR), the fibroblast growth factor receptor (FGFR), and the platelet-derived growth factor receptor (PDGFR) tyrosine kinases^43,44^. Inhibition of the signalling pathway at the kinase domain may result in the induction of endothelial cell apoptosis, reduction in tumor vasculature, tumor cell migration, and proliferation. NTD, as an anticancer agent in the treatment of non-small-cell lung carcinoma (NSCLC), was proved in clinical studies^45^. Although Nintedanib was developed and already launched as Ofev in an oral capsule formulation for the treatment of idiopathic pulmonary fibrosis (IPF), metastatic NSCLC, ovarian, prostate and colorectal cancer, the drug is also under the process of clinical trial studies in various other cancer types^46^. Since the drug has been minimally explored for the TDD application, the present work provides insights into TDD systems containing the poorly water-soluble drug NTD. We present the nanoformulation for TDD application prepared by the surface modification of MNS through monolayer coverage of APTES and subsequent loading of the anticancer drug NTD (see Scheme 1). The magnetic properties of MNS and surface modified MNS are thoroughly studied. The loading and release profile of NTD are investigated under different physicochemical conditions, and the cytotoxicity of the designed nanoformulation on the L-132 cancer cell line is also reported.

## Materials and method

### Materials

All the chemical precursors used for the synthesis of MNS were of analytical grade without any further purifications. Ferric chloride hexahydrate (FeCl_3_⋅6H_2_O), ethylene glycol (CH_2_(OH)⋅CH_2_OH), and anhydrous sodium acetate (CH_3_COONa) were procured from S D Fine-Chem India. APTES was purchased from Sigma-Aldrich. Spectroscopic grade organic solvents such as ethanol, dimethyl sulfoxide (DMSO), toluene, and methanol were purchased from Thomas Baker (Chemicals) Pvt. Ltd. Thermo Fisher Scientific India Pvt. Ltd. Cipla Ltd., Mumbai, kindly gifted the Nintedanib. Dulbecco’s modified eagle’s medium (DMEM), L-glutamine, antibiotics (streptomycin-penicillin solution), fetal calf serum (FCS), trypan blue, trypsin–EDTA, phosphate-buffered saline solution (PBS), and 3-(4,5-dimethylthiazol-2-yl)-2,5-diphenyltetrazoliumbromide (MTT) were purchased from HiMedia Laboratories. Throughout the experiments, ultra-high purity Milli Q water was used.

### Synthesis and direct functionalization of MNS by APTES

The MNS was synthesized by the solvothermal method^47,48^. Firstly, 0.6 gm of ferric chloride hexahydrate was dissolved in 30 mL ethylene glycol. Anhydrous sodium acetate (2.0 gm) was added after the complete dissolution of the iron precursor to get a yellow coloured precipitated solution, which was stirred at 50 °C for 30 min. Afterwards, the solution was transferred into a Teflon liner, fitted in a stainless-steel mould, and baked for 6 h at 200 °C in a furnace. The prepared MNS were removed from the Teflon liner and thoroughly rinsed with methanol and water in the presence of a permanent magnet and dried in a vacuum desiccator. For the functionalization of MNS by APTES, the MNS (1.0 gm) was dispersed in 100 mL methanol/toluene mixture 1:1 (V/V) by sonicating for 30 min. The resultant solution was heated at 95 °C until half of the solution was evaporated. The volume of the reaction mixture was adjusted to 100 mL by adding methanol. This procedure was repeated three times so that the solution becomes anhydrous. Then, 2% (V/V) APTES was added to the solution and kept in the shaking incubator for 24 h at 70 °C^49^. A washing step similar to that of MNS was applied to rinse the MNS-APTES.

### Ninhydrin assay

Different concentrations of leucine (20 − 100 µg/mL) in 50% ethanol (1 mL) were pipetted into a series of eppendorf tubes containing 1 mL ninhydrin solution in 2% ethanol. The above mixture was sonicated and then heated in a water bath at 100 °C for 5 min and the colour change (formation of Ruhemann’s purple) was measured at 570 nm. A suspension of MNS in 50% ethanol (4 mg/mL, stock) was prepared and used for the ninhydrin assay, as described above. The concentration of the amino groups (from APTES) on the MNS was estimated using the standard calibration curve for leucine. The assay was performed in three replicate experiments.

### Drug loading and in vitro release

The NTD loading was performed by stirring 10 mg of MNS-APTES in 10 ml ethanol with different concentrations of NTD. Details regarding the optimization of drug loading are discussed in the supplementary information. In vitro drug release study of optimum NTD-conjugated MNS was carried out at 37 °C in a 75 mL dissolution medium (pH 5.5 and pH 7.4 phosphate buffers (PBS)) in a shaking incubator for 42 h.

### Cytotoxicity assay

Early passage human lung cancer cell line L-132 was procured from National Centre for Cell Sciences (NCCS, Pune, India). The cytotoxicity assays were performed as per our previous report^50^ and briefly discussed in the supplementary information.

### Structural, elemental and magnetic characterizations

X-ray diffraction (XRD) measurements were performed on a PANalytical Empyrean diffractometer using Cu-K_α_ radiation (λ = 1.54184 Å) in the 2θ range from 10° to 80° with a step size of 0.02°. XRD patterns were analyzed by the Rietveld refinement method using the FullProf.2 k program. During refinement, the shape of the peaks was assumed to be a pseudo-Voigt function with asymmetry. The backgrounds of the patterns are fitted to a fourth-degree polynomial function. The size and shape of MNS were evaluated by a JEOL field emission scanning electron microscope (FE-SEM) JSM-7600F. A CM 20 FEG transmission electron microscope (TEM) from Philips was employed to record micrographs and energy-dispersive X-ray spectroscopy (EDX) patterns. Fourier transform infrared (FTIR) spectra were recorded at room temperature in the range of 400–4000 cm^−1^ using a Shimadzu FTIR spectrophotometer. The magnetic properties of the MNS and MNS-APTES were investigated using superconducting quantum interference device-vibrating sample magnetometry (SQUID-VSM) from Quantum Design. The magnetic hysteresis loop, zero-field cooled (ZFC), and field cooled (FC) measurements were performed over a temperature range of 2–400 K and a magnetic field up to 60 kOe. The experimental procedure for thermoremanent magnetization (TRM) measurements consisted of cooling the sample down to 2 K in the presence of a 40 kOe magnetic field, followed by magnetization measurements at increasing temperatures. For each data point at a given temperature, the sample was magnetized in the magnetic field of 40 kOe for 5 min. Afterward, the field was switched off, and the sample was allowed to relax for 1 min, followed by a magnetization measurement. Thus, the recorded magnetization is the measure of remanent magnetization of all the MNS in the blocked state at a given temperature. The X-ray photoelectron spectroscopy (XPS) analysis was performed with an ESCALAB 250Xi photoelectron spectrometer from Thermo Scientific in an ultra-high vacuum (UHV) using a monochromatic Al-K_α_ (1486.6 eV) X-ray source and a beam diameter of 300 µm. The hemispherical electron deflection analyzer operated in the constant analyzer energy (CAE) mode at pass energy of 200 eV for survey spectra and 20 eV for high-resolution spectra. The binding energies of all spectra were referenced to the binding energy of C1s (284.4 eV).

## Results and discussion

### Size and phase analysis

**Figure 1:**
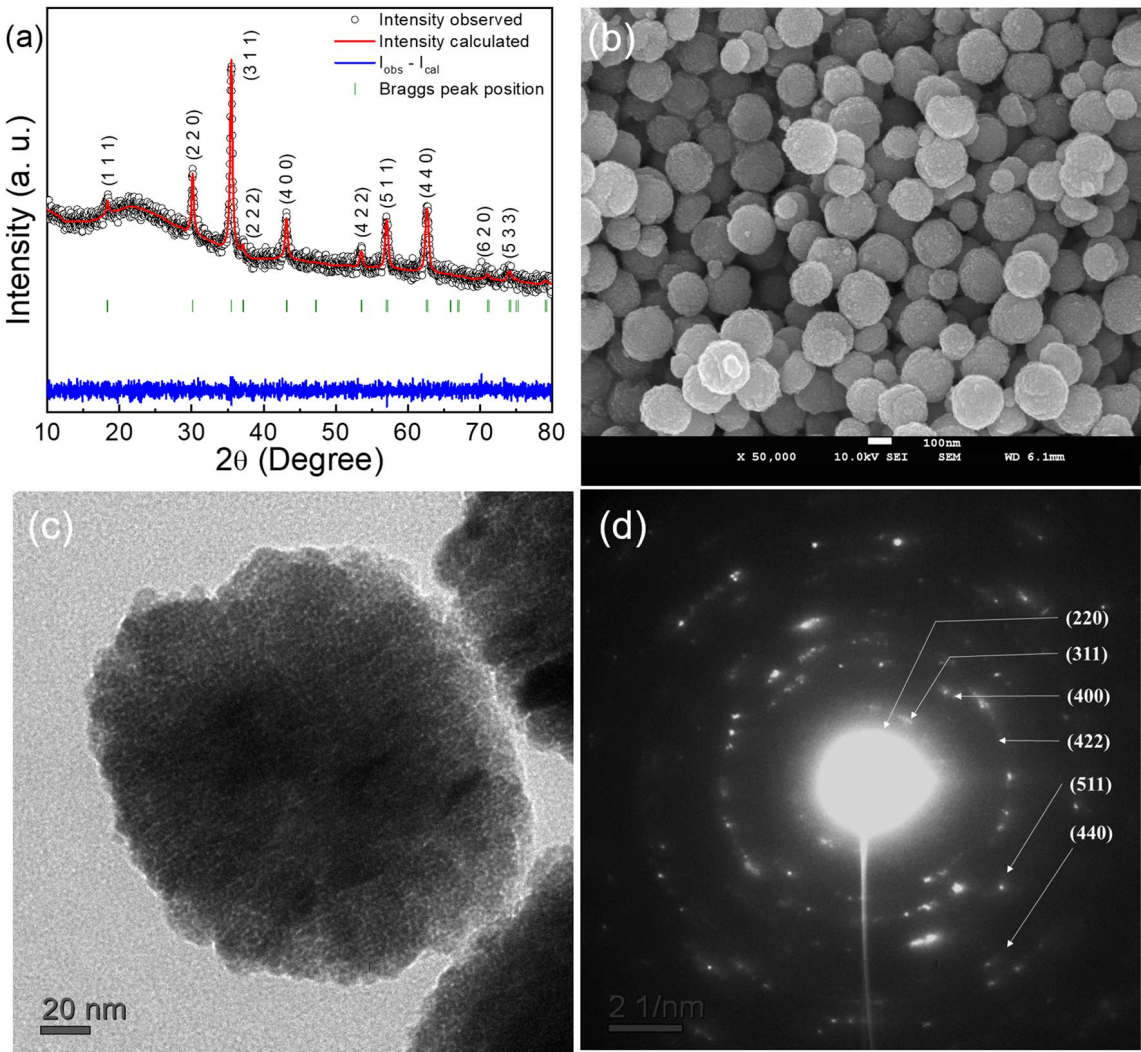
The XRD pattern and the Rietveld refinement profile using the Fullprof program (**a**), FE-SEM micrograph (**b**), TEM micrograph (**c**), and SAED pattern of MNS (**d**).

The structural and morphological investigations of MNS were performed using XRD, FE-SEM, and HR-TEM. The XRD pattern with Rietveld refinement of an MNS is shown in Fig. 1a. According to the refinement data, the MNS exhibits pure magnetite (Fe_3_O_4_) phase. The characteristic diffraction peaks for the lattice planes (220), (311), (400), (422), (511), and (440) of a cubic structure with space group Fd-3m were observed (JCPDS card No. 01-088-0866). The detailed parameters extracted after the refinement of diffraction spectra are tabulated in Table S1. The lattice parameter 8.3852 Å and the unit cell volume 589.58 Å^3^ are comparable with the reference value for stoichiometric magnetite. The average crystallite size determined by the Debye–Scherrer formula for the most intense peak (311) was about 23 nm. Representative FE-SEM and TEM micrographs displaying the spherical shape of MNS are presented in Fig. 1b,c, respectively. It can be seen from these micrographs that the spheres consist of smaller particles. The size distribution histograms are drawn to estimate the average diameter of MNS (Fig. S1a) and constituent particles (Fig. S1b) using multiple FE-SEM and TEM micrographs, respectively. The average size of the MNS is about 180 nm, and that of constituent particles is about 27 nm. The SAED pattern of the MNS (Fig. 1d) has concentrated rings comprising small spots indicating its polynanocrystalline nature. All diffraction rings correspond to the magnetite phase, which corroborates well the XRD results. It has previously been suggested that the size and shape of nanostructures may determine the biodistribution of particles^51^. Decuzzi et al*.*^52^ studied NPs in the range of 50 nm–10 μm and observed that particles having a size less than 500 nm support the Brownian motion and can have colloidal stability. Depending on application perspectives, different sizes and shapes of NPs are recommended. The particles having spherical morphology and critical size less than 200 nm can be used for TDD applications^53–56^.

### FTIR analysis

**Figure 2:**
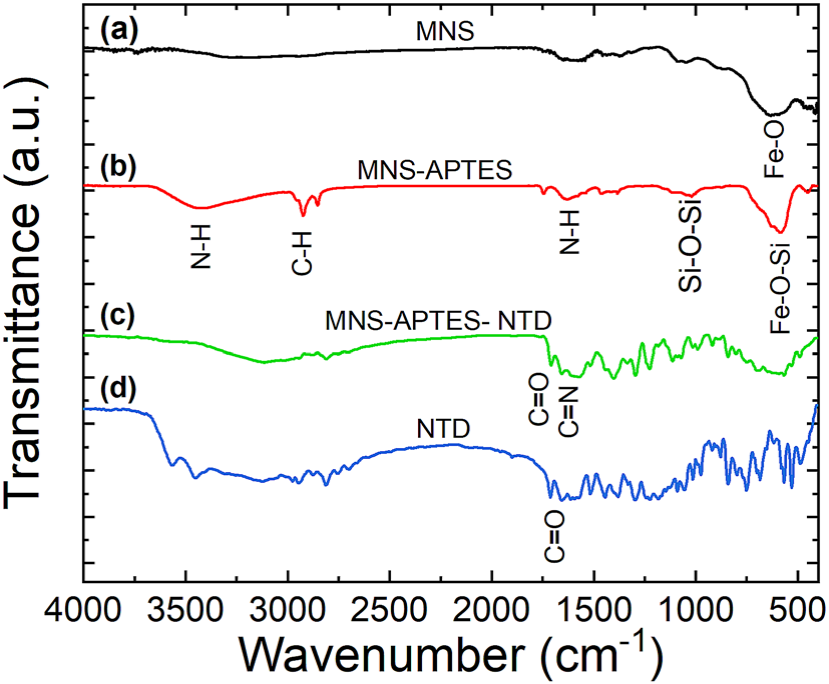
FTIR spectra of (**a**) MNS, (**b**) MNS-APTES, (**c**) MNS-APTES-NTD, and (**d**) NTD.

The FTIR spectra of MNS, MNS-APTES, NTD conjugated MNS, and NTD are shown in Fig. 2a–d, respectively. The strong absorption peak at 589 cm^−1^ assigned to Fe–O stretching vibrations from iron oxide can be observed for MNS, MNS-APTES, and MNS-APTES-NTD^57^. Additionally, the spectrum in Fig. 2b may contain a stretching vibration of Fe–O–Si at 589 cm^−1^ overlaying with Fe–O vibrations from iron oxide^50^. The peak corresponding to Si–O–Si at 1015 cm^−1^, and the bending and stretching vibrational modes of NH at 1637 cm^−1^ and 3400 cm^−1^, respectively, confirm the coverage of silane with a free amine group of APTES on the MNS surface^58,59^. A band observed at 2920 cm^−1^ is due to C-H stretching vibrations of the anchored propyl group^60,61^. The TEM–EDX spectra (see Fig. S2 in supplementary information) of the APTES modified MNS also shows the presence of silicon (Si) confirming the APTES coverage on the surface of MNS. APTES coverage on the MNS surface provides functional amino groups for drug conjugation. The conjugation of NTD on MNS-APTES was confirmed by the appearance of the strong absorption peak at 1653 cm^−1^ attributed to the C = N stretching vibration of the imine bond formed between the carbonyl group of NTD and amine group of MNS-APTES^62,63^. The characteristic bands appearing in Fig. 2d at 2938 cm^−1^, 1706 cm^−1^, 1653 cm^−1^, 1508 cm^−1^, 1435 cm^−1^, and 1280 cm^−1^ corresponding to C-H stretching (CH_3_), C = O stretching (from ester), and C = O stretching (from amide) in the NTD are also observed in Fig. 2c for MNS-APTES-NTD^64^. This appearance of characteristic NTD bands in MNS-APTES-NTD nanoformulation indicates the successful conjugation of drugs.

### Monolayer coverage

The quantification of the amine groups existing on the MNS was evaluated by ninhydrin colourimetric assay. The nucleophilic displacement reaction among the primary amines and ninhydrin occurs, which includes the displacement of a hydroxyl group from ninhydrin by amine. It produces a colour complex known as Ruhemann's purple. The free − NH_2_ groups estimated were found to be around 41.5 ± 3 µmol/g of MNS. The calculation of fractional monolayer coverage was performed as reported by Sun et al*.*^65^, assuming four aminopropylsilanes/nm^2^ for monolayer coverage of APTES and using the surface area (6.44 m^2^/g) calculated from the mean MNS diameter (180 nm). The details regarding the calculation of surface area (m^2^/g) of MNS is provided in SI.

1 $$\mathrm{Fractional\,monolayer\,coverage}=\frac{\mathrm{total\,amine\,from\,ninhydrin\,assay}}{\mathrm{estimated\,amine\,for\,monolayer\,coverage}} \times 100\mathrm{\%}$$

2 $$= \frac{41.5 \times {10}^{-6} (\mathrm{mol}/g)\times 6 \times {10}^{23} (\mathrm{mol})}{4 (/{nm}^{2}) \times surface area ({nm}^{2}/g) } \times 100\%$$

3 $$\mathrm{Fractional\,monolayer\,coverage}=96.6\%$$

These results reveal monolayer coverage of APTES over MNS. It was reported that primary amine-modified monolayers on the NPs surface significantly improved the cellular delivery^66^. Besides, the monolayer coverage can overcome the problems associated with polymerization of the initial silane, possible formation of heterogeneous multilayers on the surface in the presence of excess silane and the lack of stability of the aminated surfaces^67^.

### XPS analysis

**Figure 3:**
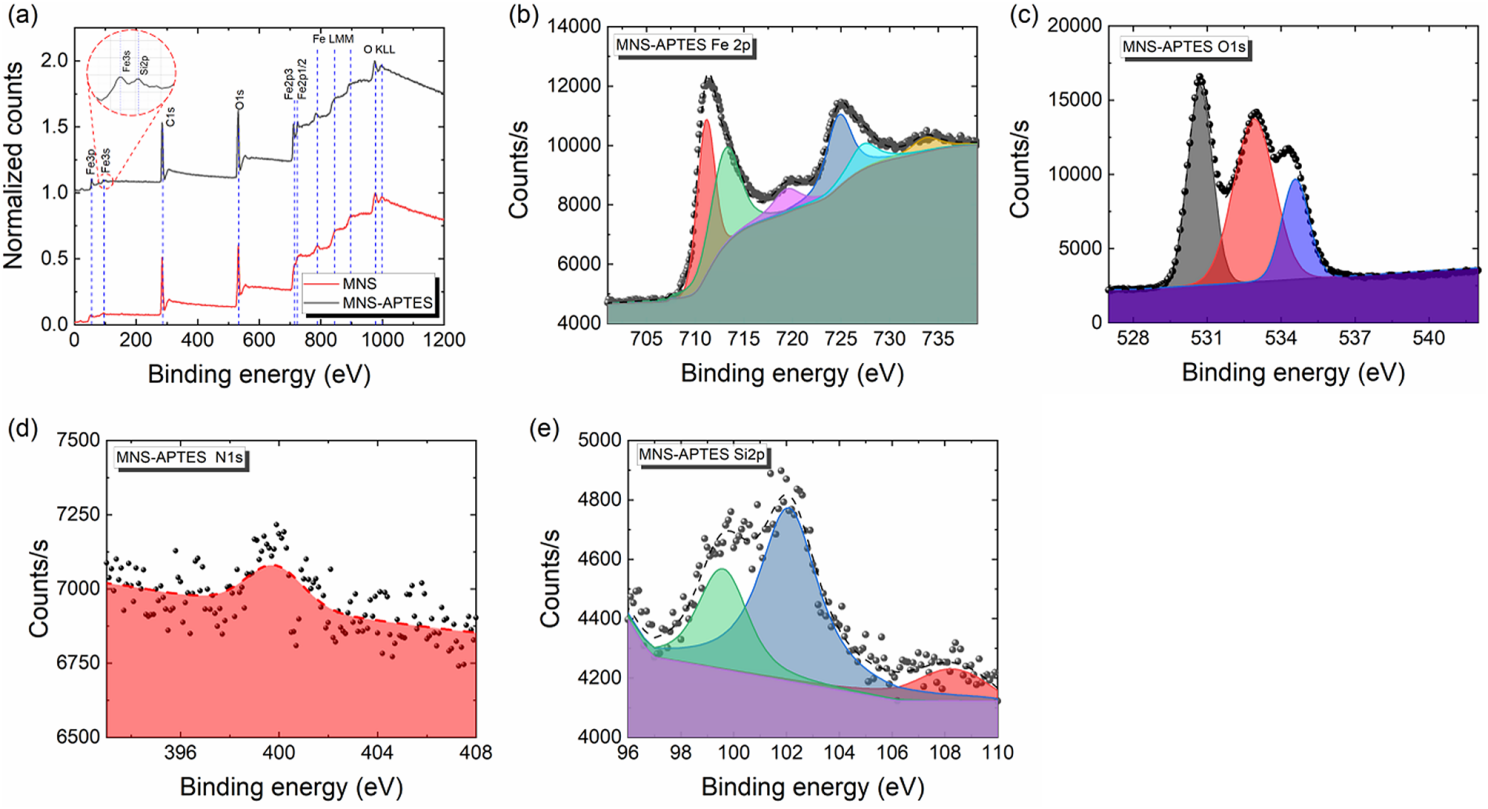
(**a**) XPS survey spectra of bare and APTES MNS. The observed spectral features are labelled with relevant elemental peaks. The regions for (**b**) Fe(2p), (**c**) O(1 s), (**d**) N(1 s), and (**e**) Si(2p) for the APTES coated MNS.

The surface electronic states and the chemical composition for MNS and MNS-APTES were probed by XPS. The survey spectra (Fig. 3a) of the MNS reveals the presence of Fe, O, C, and an additional peak of Si in the case of MNS-APTES. No clear peak for N was observed in the survey of MNS-APTES due to the smaller XPS cross-section of N1s in comparison to Si2p and other core-levels. Nevertheless, a clear peak at 399.1 eV arising from N1s in the amine groups was observed in the high-resolution spectrum (Fig. 3d)^68^. The high resolution XPS spectrum of Fe2p (Fig. 3b) shows the Fe2p_3/2_ peaks of Fe^2+^ and Fe^3+^ states located at 711.1 eV and 713.2 eV, respectively. This agrees with the values reported in the literature^69,70^, indicating the presence of Fe_3_O_4_. The Fe2p_3/2_ main peak with a satellite peak on the higher binding energy side at 719.4 eV additionally indicates the iron to be in the + 2-oxidation state. The spectral features at 724.8, 727.3, and 733.7 eV are the Fe2p_1/2_ components of the corresponding spin–orbit doublets^70^. The high-resolution deconvoluted O1s XPS spectra (Fig. 3c) for MNS-APTES samples show a typical oxygen peak at 529.5 eV corresponding to magnetite lattice oxygen (O^2−^)^70^. The peaks at 533.4 eV and 531.7 eV can be assigned to oxygen associated with SiO_2_^71^ and surface hydroxyl or carboxyl groups (OH/CO) on MNS-APTES^72^. The presence of the SiO_2_ peak in the Si2p spectrum at 102.6 eV and Si–C at 100.2 eV (Fig. 3e) is consistent with the development of interlinking silane molecules on the MNS surface^73^.

### Magnetic characterization

**Figure 4:**
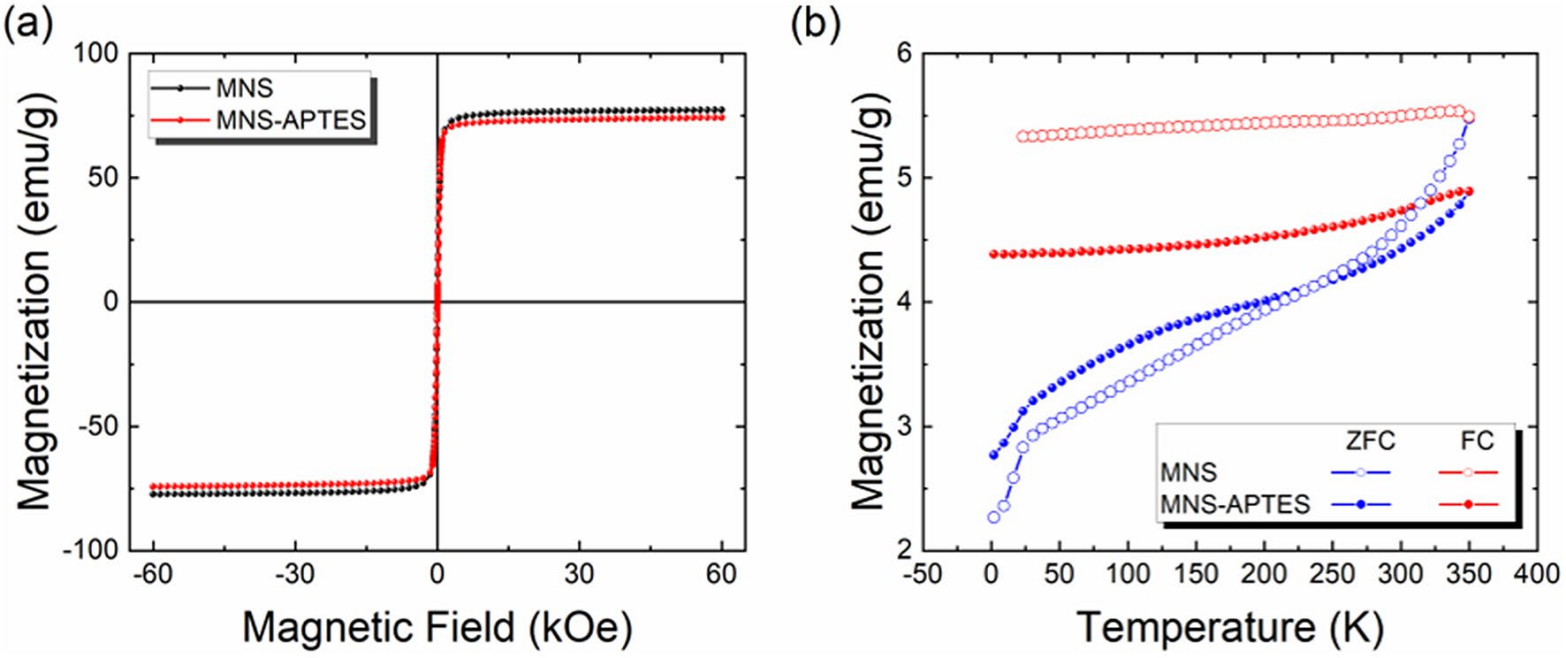
(**a**) Room-temperature M-H curves of MNS and MNS-APTES and (**b**) temperature-dependent FC-ZFC magnetization curves of MNS and MNS-APTES.

The magnetic properties of bare MNS and APTES functionalized MNS was evaluated by measuring the M-H curves at room temperature (300 K), as shown in Fig. 4a. The room temperature M-H curves unveiled the superparamagnetic behaviour for both samples, as the curve passes through the origin and has nearly zero coercivity^74^. The saturation magnetization values (M_S_) of MNS and MNS-APTES are about 77 emu/g and 74 emu/g. The observed M_S_ values are smaller than the corresponding bulk value (92 emu/g), which is attributed to surface effects in MNS^75^. A relatively small or negligible reduction in the saturation magnetization M_S_ value of MNS was observed after APTES functionalization. The nominal reduction in M_S_ after functionalization is occurred due to the monolayer coverage of APTES on the MNS surface. The temperature-dependent magnetic behaviour was investigated by ZFC–FC measurements (Fig. 4b). The FC curves for both samples exhibit a plateau, suggesting a strong dipolar interaction among particles^76,77^. This also explains the quasi-linear thermal dependence of the ZFC curve with a subtle increase in magnetization. In such cases, it is difficult to estimate the blocking temperature from ZFC–FC measurements.

**Figure 5:**
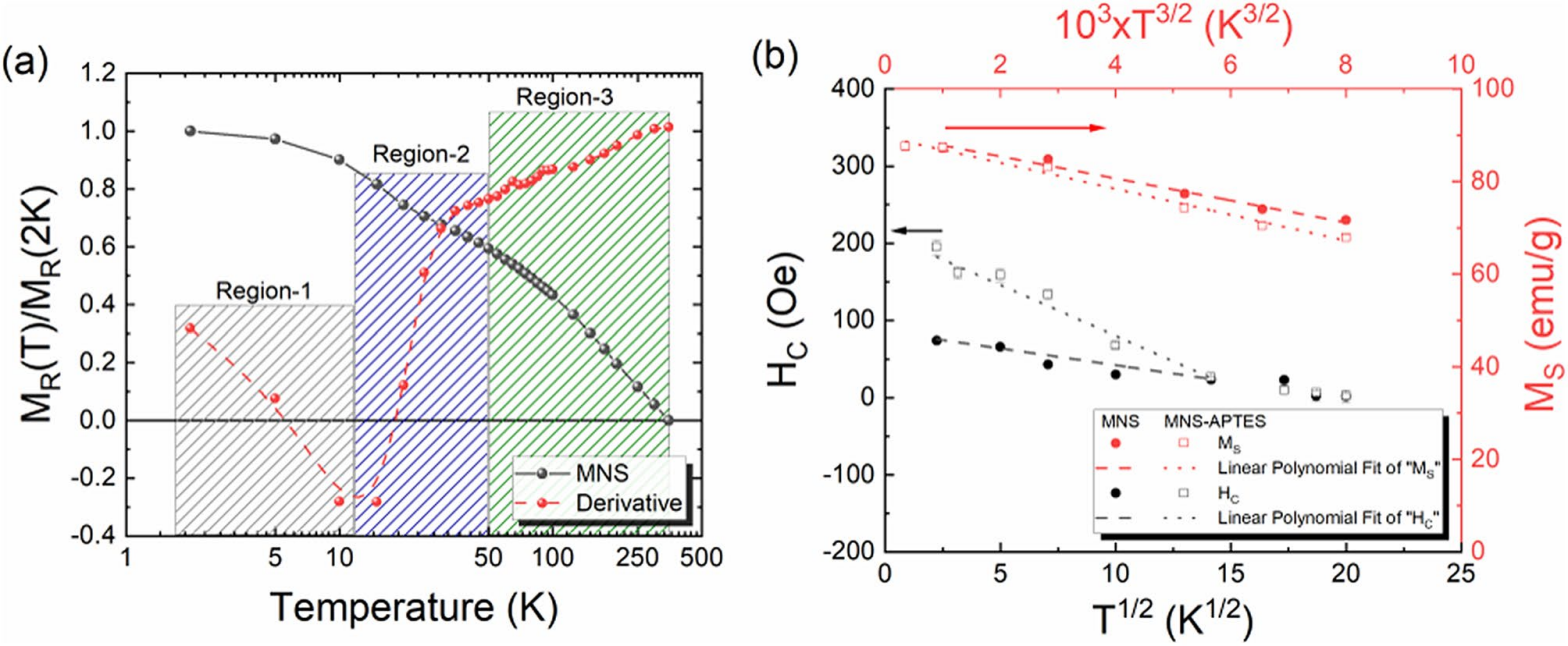
(**a**) The thermoremanent magnetization (black circles) and its first-order derivative (red circles) as a function of temperature, (**b**) The coercive field (H_C_) vs. T^1/2^ (left vs. bottom axis) and saturation magnetization (M_S_) vs. T^3/2^ (right vs. top axis) for MNS (circles) and MNS-APTES (squares).

To get the information on the magnetic phases present in the samples by thermoremanent magnetization, several hysteresis loops were recorded at different temperatures. The normalized thermoremanent magnetization (TRM) values measured for MNS are shown in Fig. 5a, which helps to find the magnetic phase transition temperature for the smaller MNPs constituting the MNS. As anticipated for NPs, a gradual decrease in remanent magnetization was observed with increasing temperature for both MNS and MNS-APTES (not shown here). The thermoremanent magnetization curve reveals a subtle step at around 10–40 K, which is similar to the feature observed below 50 K in the field cooled curve (Fig. 4)). To highlight the changes in TRM variation, the first-order derivative of the TRM curve with respect to temperature is plotted, which reflects the distribution of the blocking temperature^78^. The derivative curve can be divided into three regions depending on the observed change in slopes, namely region-1 (T < 10 K), region-2 (10 K < T < 50 K), and region-3 (T > 50 K). Region-1 for the range T < 10 K corresponds to extremely fine MNPs with blocking temperatures below 10 K still being in the paramagnetic state. Region-2, between 10 and 50 K, represents particles with a moderate size, which constitute the majority of the sample. The third region T > 50 K is for the MNPs in the samples with blocking temperature considerably above room temperature.

Figure 5b presents the magnetization (M_S_) and coercive field (H_C_) response of MNS and MNS-APTES. MNS has a higher saturation magnetization compared to MNS-APTES, which scales linearly with increasing temperature. The scaling of M_S_ with temperature can be explained using Bloch’s law^79^, which is related to low energy collective spin wave excitations (magnons).

4 $${M}_{S}(T)={M}_{S0}\left[1-{\beta \left(T\right)}^\frac{3}{2}\right]$$

Here, M_S_(T) and M_S0_ are the saturation magnetization at any given temperature (T) and absolute zero temperature (0 K), respectively. “β” is the Bloch constant, which depends on the Curie temperature (β ∝ 1/T_C_). The values of M_S0_ of 90.2 emu/g and 89.7 emu/g were obtained for MNS and MNS-APTES, respectively.

In general, MNS exhibits hysteretic behaviour for the temperature below the blocking temperature and non-hysteretic behaviour above the blocking temperature. The coercive field (H_C_) is observed to decrease monotonously with the square root of temperature and reaches zero at blocking temperature. The temperature dependence of H_C_ can be expressed with Kneller’s law^80,81^,

5 $${H}_{C}(T)={H}_{c0}\left[1-{\left(\frac{T}{{T}_{B}}\right)}^\frac{1}{2}\right]$$

Here, T_B_ is the blocking temperature, and H_C0_ is the coercive field at 0 K. The value for H_C0_ was obtained by linear fitting the H_C0_ vs T^1/2^ plot in the temperature range between 5 and 400 K. The values of H_C0_ obtained for MNS and MNS-APTES were 92.5 Oe and 211.6 Oe, respectively. Here it is worth mentioning that only a limited temperature range was chosen for the linear fitting, as the accuracy of H_C_ is drastically lower above the blocking temperature. The estimated blocking temperatures for MNS and MNS-APTES were 126 K and 346 K, respectively. A probable reason for the higher value of T_B_ estimated from the Eq. 5 is because the investigated samples are conglomerate of various sized interacting NPs. This assumption is further supported by the ZFC–FC (*cf.* Fig. 4b) and TRM results (*cf.* Fig. 5a). Nevertheless, the linear fit to H_C_ vs. T^1/2^ and a negligibly small value of H_C_ below 200 K (14 K^1/2^) indicates superparamagnetic response.

### NTD loading and release

**Figure 6:**
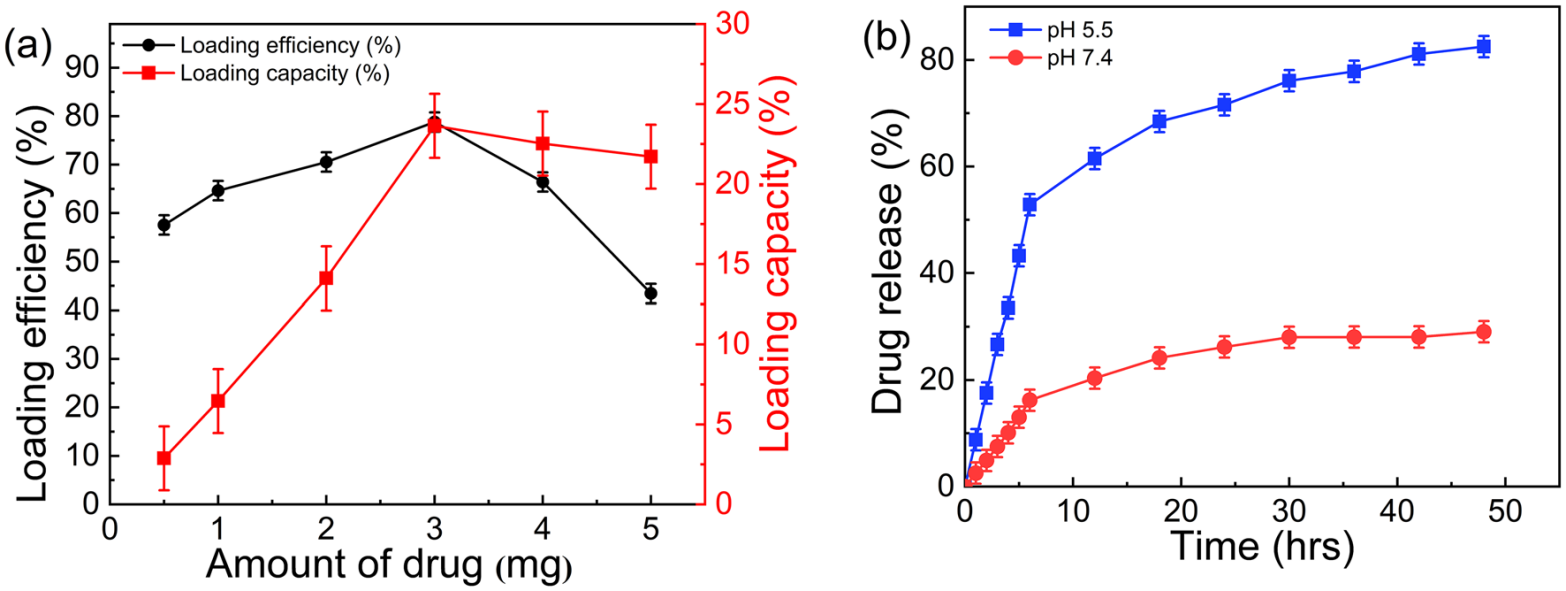
The loading and release of NTD (**a**) Drug loading efficiency and drug loading capacity when 1 mg MNS was reacted with different amounts of NTD, (**b**) Drug release from MNS-APTES-NTD nanoformulation at pH 7.4 and pH 5.5 in PBS (37 °C).

To achieve optimum drug loading on MNS, the ratio of MNS:NTD was varied. The drug loading efficiency and loading capacity for different MNS:NTD ratios are summarized in Table S2 and shown in Fig. 6a. With a fixed MNS weight, initially, the NTD loading increased linearly with an increase in the amount of NTD and saturated for higher NTD amount. For MNS:NTD ratio (1:3), a maximum loading efficiency (79%) and loading capacity (23%) corresponding to greater conjugation of NTD was observed. This optimum nanoformulation (MNS:NTD, 1:3) was used for further studies.

**Scheme 2:**
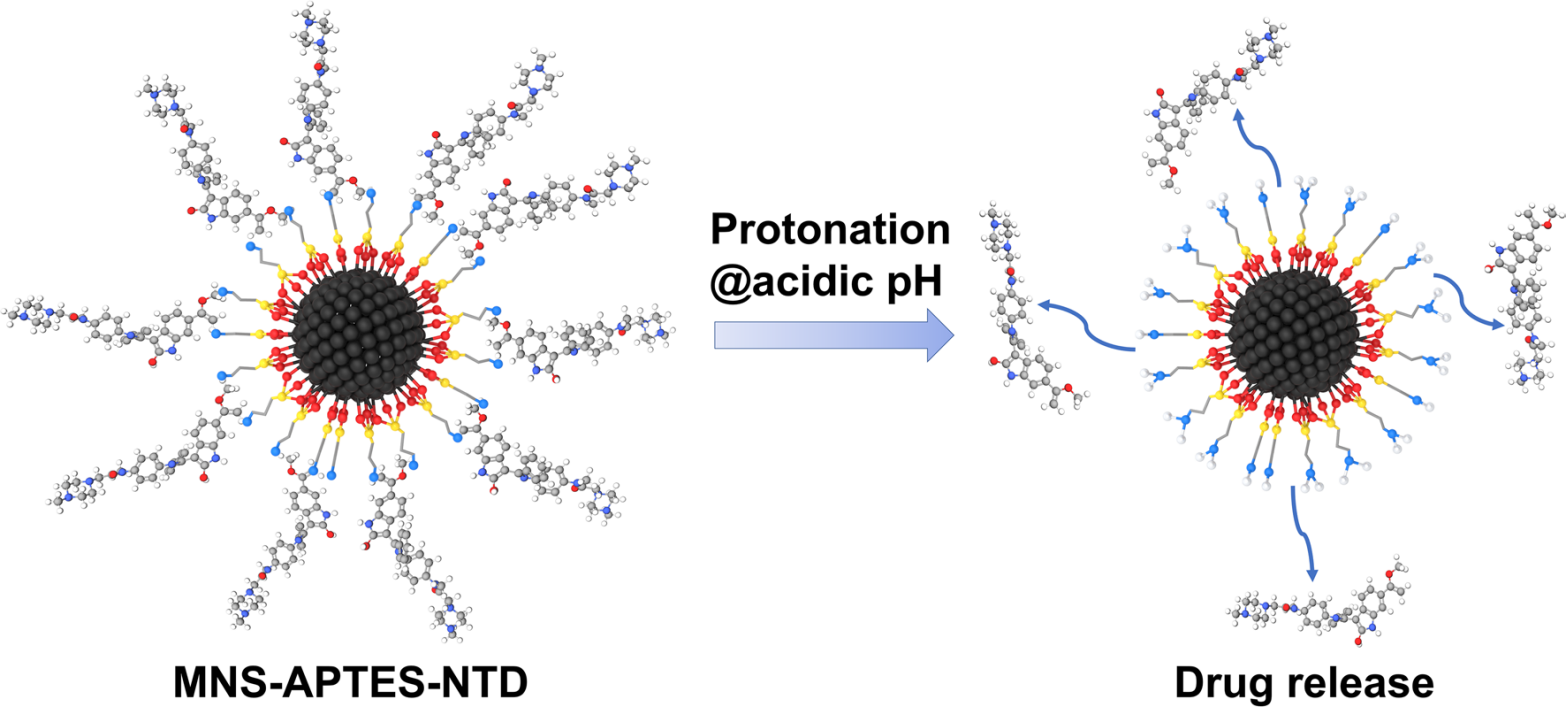
Protonation and release of NTD from the MNS-APTES-NTD nanoformulation.

The drug release from the MNS-APTES-NTD nanoformulation at 37 °C at pH 7.4 and 5.5 is shown in Fig. 6b. The pH values used to investigate the drug release mimics the physiological and the endosomal pH value of cancer cells. At physiological pH 7.4, the nanoformulation was quite stable, and only 28% cumulative drug release was observed after 48 h. The low drug release profile at neutral pH is observed due to the stable imine bond between MNS-APTES and NTD at physiological conditions. At acidic pH 5.5, the cumulative NTD release was about 50% within the first few hours, and release was up to 85% in 48 h. The mechanism of the controlled drug release can be attributed to the cleavage of the pH-responsive imine bond between NTD and functionalized MNS. Scheme 2 shows the possible mechanism of drug release in which imine groups get protonated under acidic conditions.

The functional polymers which exhibit ionizable groups (i.e., − NH_2_, − COOH, − SO_3_H, − PO_3_H_2_, − B(OH)_2_) at support may get converted into charged moieties depending on the different pH conditions^82^. The linkages like imine bond are mostly stable under physiological conditions (pH ≈ 7.4), whereas they get hydrolyzed in acidic surroundings. The observed different pH response is ascribed to the protonation state of terminal primary amino groups under different pH conditions^83^.

### In vitro cytotoxicity study

**Figure 7:**
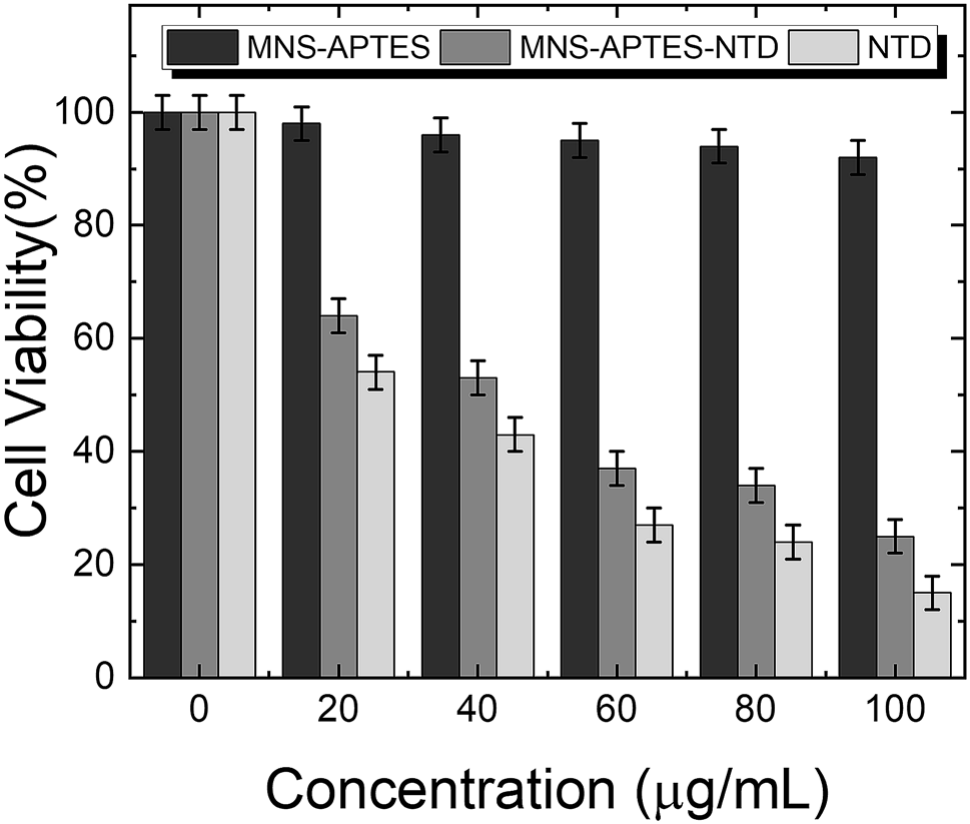
In vitro L-132 cancer cell viability after exposure to MNS-APTES, MNS-APTES-NTD, and free NTD at different concentrations.

The MTT assay was performed to examine the in vitro cytotoxicity of MNS-APTES, MNS-APTES-NTD, and free NTD. Figure 7 shows the cell viability of L-132 cancer cells after exposure to all the samples at various concentrations (20–100 μg/mL). Even at higher levels of MNS-APTES, the survival rate of L-132 cells does not grieve due to the biocompatibility of surface-modified MNS. The viability of L-132 cells decreased with an increase in concentrations of free NTD and MNS-APTES-NTD, indicating dose-dependent cytotoxicity of the formulations. The free NTD and MNS-APTES-NTD showed cellular toxicity of 57% and 47% at 40 μg/ml, respectively. For 100 μg/ml of free NTD and MNS-APTES-NTD, cellular death reached 85% and 75%, respectively. The difference in cell viability of MNS-APTES-NTD and free NTD can be attributed to the lower amount of NTD in the nanoformulation compared to free NTD. Similar cytotoxicity observed for NTD loaded nanoformulation, and free NTD suggests that NTD released from the nanoformulation has the same anticancer activity as free NTD.

The docking studies were performed among FGFR-4 and NTD; they reveals that amino acid residue Met524 involves in the binding of NTD with the protein. Further detailed surface representation of the docked complex and discussion is given in Table S3, Fig S3 and Fig S4.

## Conclusions

This work demonstrates the pH-responsive, controlled release of poorly water-soluble model drug NTD from the nanoformulation prepared by monolayer coverage of APTES over MNS. The use of a direct functionalization strategy and monolayer coverage of APTES prevents the loss in the magnetization of MNS reported for several functionalization approaches^30–32,42,84^. Thermoremanent magnetization studies reveal the superparamagnetism and qualitative distribution of magnetic properties in the MNS. Functionalization of APTES and loading of the anticancerous drug NTD is confirmed by FTIR. A drug loading capacity of 23.6% is obtained. Monolayer coverage of aminosilane is established by quantifying the amine groups with the ninhydrin assay. NTD is conjugated with MNS-APTES through the acid liable imine bond. At physiological conditions, the MNS-APTES-NTD nanoformulation maintains high stability and inhibits drug release. In contrast, the cleavage of imine bonds in an acidic environment would lead to the drug release on demand. The MNS-APTES-NTD nanoformulation exhibits dose-dependent cytotoxicity for the L-132 cell line. Further magnetic studies of the MNS with a different size distribution of the constituent particles are underway to investigate the interparticle interaction and to explore them for various applications. The higher magnetization of functionalized MNS owing to monolayer coverage can be useful in many nanobiotechnology applications such as magnetoresistive biosensors, nanobiocatalysis, magnetic cell separation, etc.

## Supplementary Information

Supplementary Information
