# Supplementary material for: APTES monolayer coverage on self-assembled magnetic nanospheres for controlled release of anticancer drug Nintedanib
Source: Sci Rep. 2021 Mar 11;11:5674. doi: 10.1038/s41598-021-84770-0 (PMC7952395; doi:10.1038/s41598-021-84770-0)
Supplement: Supplementary file 1 — Supplementary Information [file 41598_2021_84770_MOESM1_ESM.docx]

***Supplementary Information***

**APTES monolayer coverage on self-assembled magnetic nanospheres for controlled release of anticancer drug Nintedanib**

V.C. Karade^1,2†^, A. Sharma^3†^, R.P. Dhavale^1,4^, R.P. Dhavale^5^, S. R. Shingte^6^, P.S. Patil^1,7^, J. H. Kim^2^, D.R.T. Zahn^3^, A.D. Chougale^8^, G. Salvan^3,*^, and P.B. Patil^6,*^

^1^School of Nanoscience and Technology, Shivaji University, Kolhapur, Maharashtra-416004, India.

^2^Optoelectronic Convergence Research Center and Department of Materials Science and Engineering, Chonnam National University, Gwangju, 500-757, South Korea.

^3^Semiconductor Physics, Chemnitz University of Technology, D-09107 Chemnitz, Germany.

^4^Department of Materials Science and Engineering, Yonsei University, Seoul-03722, South Korea.

^5^Department of Pharmaceutics, Bharati Vidyapeeth College of Pharmacy, Kolhapur, Maharashtra 416013, India.

^6^Department of Physics, The New College, Shivaji University, Kolhapur, Maharashtra-416012, India.

^7^Department of Physics, Shivaji University, Kolhapur, Maharashtra-416004, India.

^8^Department of Chemistry, The New College, Shivaji University, Kolhapur, Maharashtra-416012, India.

^*^**Corresponding author**:

G. Salvan (email: [salvan@physik.tu-chemnitz.de](mailto:salvan@physik.tu-chemnitz.de) ); P.B. Patil (email: [prashantphy@gmail.com](mailto:prashantphy@gmail.com) )

**†** V.C.K. and A.S contributed equally to this work

**Drug loading and in vitro release**

**a) Drug loading**

The drug loading was achieved by dispersing 10 mg MNS-APTES in 10 ml ethanol. Various concentrations of NTD (0.5 mg/mL, 1.0 mg/mL, 2 mg/mL, 3 mg/mL, and 5 mg/mL) were prepared in ethanol wherein DMSO added was not more than 5%. The prepared NTD solution was added dropwise in dispersed MNS-APTES to maintain functionalized MNS:NTD ratios (1:0.5, 1:1, 1:2, 1:3, 1:4, 1:5) as batches A, B, C, D, E, and F respectively. For the chemical conjugation, 2M ethanolic sodium hydroxide solution was added in the above mixture, sonicated for 15 min and stirred for 10 h at room temperature. The MNS-APTES-NTD were separated by a magnet and washed several times with distilled water and dried in a vacuum desiccator. The total amount of NTD in the supernatant was measured by the spectroscopic method at 391 nm. The percent loading efficiency was calculated as the quotient of the difference between the initial drug concentration and drug concentration in the supernatant (drug loss) to the initial concentration of the drug.

|  | $Loading efficiency \% = \frac{Initial drug concentration-drug loss}{Initial drug concentration} \times100$ | 1 |
| --- | --- | --- |

The percent loading capacity (drug content) of MNS was calculated by the following equation,

|  | $Loading capacity \%= \frac{mg (drug-loaded)}{mg (MNS)} \times100$ | 2 |
| --- | --- | --- |

Where mg_(drug loaded)_ is the quantity of the drug loaded on the MNS and mg_(MNS)_ is the gross quantity of the MNS.

**b) *In vitro* drug release**

The *in vitro* drug release study of NTD-conjugated MNS was carried out at 37°C in a 75 mL dissolution medium (pH 5.5 and pH 7.4 phosphate buffers) in a conical flask under constant shaking for 42 h. The weighted quantity of optimized Batch D (drug amount 10 mg, equivalent to 23.64% of loaded MNS) was exposed to a 75 mL dissolution medium in a conical flask and placed in an incubator shaker at 75 rpm. To determine drug release, 5 mL of solution was removed at specific fixed time intervals and was replaced with 5 mL of fresh buffer solution to consistently maintain the volume of dissolution media. The cumulative drug release was calculated by the equation

|  | $Cumulative release \%= \frac{mg(drug released)}{mg(total drug)}\times100$ | 3 |
| --- | --- | --- |

Where mg (drug released) is the amount of drug released at the time t, and mg (total drug) is the total amount of drug present in drug-loaded MNS.

**Cytotoxicity**

Early passage human lung cancer cell line L-132 was procured from National Centre for Cell Sciences (NCCS, Pune, India) in the form of adherent culture into 75 cm^2^ culture flasks using, Dulbecco’s Modified Eagle media (DMEM) as the base medium, supplemented with 10% heat-inactivated FCS, 2 mM L-glutamine. The 1% antibiotics (10 mg/mL streptomycin and 100 U/mL penicillin G) was maintained at 37°C with 5% CO_2_, 95% air in a humidified incubator (Eppendorf, New Brunswick Galaxy 170S). Before performing the assay, cells were subcultured splitting in ratio as 1:2 to 1:8 to get subconfluent culture (70-80%). The subcultured cells were seeded at 1×10^6^ cells/ml using 0.25% trypsin, 5% CO_2_ at 37°C. Furthermore, the cells were observed for nearly 80% confluency, and the culture media was removed from the flask. The cells were briefly rinsed with 10 mL pH 7.4 phosphate buffer solution (PBS). The volume of trypsin-EDTA, 0.25% (wt/vol) was uniformly added to cover the cell growth surface. The specified volume (10 mL) of culture medium containing FCS was added when the cells were observed as dissociated, to obtain a homogenous cell suspension. The concentration of cells was counted by using a 1:1 mixture of cell suspension and 0.4% (wt/vol) trypan blue solution in a haemocytometer chamber under an inverted phase microscope (Fusiontek, Ahmedabad).

The MTT [3-(4,5-dimethylthiazol-2-yl)-2,5-diphenyltetrazoliumbromide] assay was utilized to determine the cytotoxicity of MNS-APTES, MNS-APTES-NTD, and free NTD using 96 well microtiter plates. L-132 cells were briefly seeded in 96-well plates at 2×10^4^ cells/mL in serum DMEM. After 24 h the medium was replaced with serum-free DMEM and incubated to acquire proper confluent cell layer. The seeded cells were treated with gradient concentrations of MNS-APTES, MNS-APTES-NTD, and free NTD (20-100 μg/mL) at 37°C for 48 h. After the incubation period, 20 µL of MTT (0.5 mg/mL) was added to each well and plates were incubated in a CO_2_ incubator for an additional 3 h at 37°C. The blue-violet formazan crystals formed after cell lysis was dissolved by adding 150 µL DMSO. The intensity of colour was measured at 570 nm using a microplate reader (Bio-Rad, India).

**Docking analysis of FGFR-4 with Nintedanib**

Molecular docking studies were performed using Molecular Operating Environment (MOE)-2015 on a computer having Intel(R) Core i3 2.10 GHz workstation ^S1^. The crystal structure of the protein complex with native ligand Ponatinib (PDB id 4UXQ) was taken from the RCSB protein data bank ^S2^. Docking steps were initiated as per the protocol of software; briefly, the FGFR-4 protein (PDB id 4UXQ) was imported in the MOE interface, the native ligand was removed from the protein, hydrogen atoms were added along with geometry optimization followed by energy minimization using the MMFF94 forcefield. Default parameters as required for systematical confirmation was set for the resulting model with an RMS gradient of 1 cal/mol.

The protein was allowed for the identification of active binding sites, dummy atoms were created, and the backbone and residues from the resulting alpha spheres were kept fixed, followed by energy minimization. Similarly, the ligand named Nintedanib was retrieved from Pubchem (CID:135423438) and loaded in the MOE interface in 2D form. The ligand was prepared for partial charges and energy minimized using the MMFF94 forcefield. The docking process was initiated by setting an appropriate receptor and ligand. After running ten different conformations, the best docking poses were selected based on the root mean square deviation (RMSD) values and the binding affinity score called the docking score (S-score). The resulting docked poses were compared with the native ligand and were chosen for further protein-drug docking analysis. Binding energies and bond distances of the docked complex were calculated from ligand interaction studies at the active binding site of the receptor protein.

**Table S1.** Detailed parameters of MNS crystal structure extracted from Rietveld refinement

| **Chemical formula** | **Fe_3_O_4_** |
| --- | --- |
| Crystal system | Cubic |
| Space Group | F d-3m |
| Z | 8 |
| *a* (Å) | 8.3852(5) |
| V (Å^3^) | 589.619(7) |
| R_wp_ | 1.23 |
| R_p_ | 1.54 |
| Res | 1.49 |
| *χ*^2^ | 1.07 |
| GOF | 1.0 |
| 2*θ* range (°) | 10-80 |
| 2θ step width (°) | 0.02 |
| Number of reflections | 19 |
| crystallite size (nm) | 23(1) |

The numbers in brackets indicate errors of the last digit.

| **** | **** |
| --- | --- |
| **Figure S1.** The average size distribution of a) MNS and b) constituent MNP | |
| 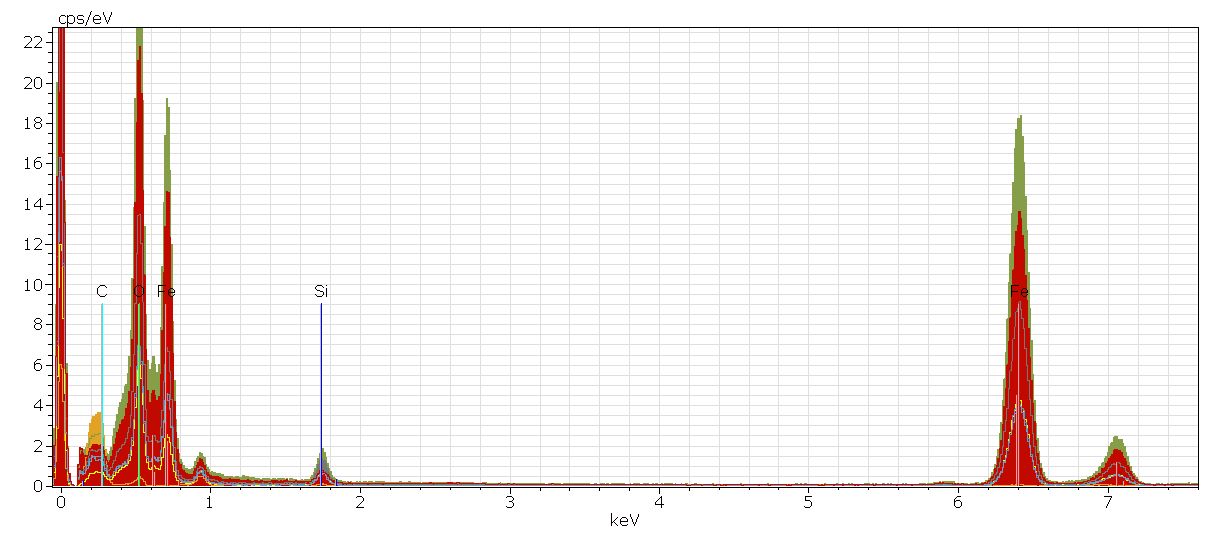 | |
| **Figure S2.** EDX spectra of APTES modified MNS   - The calculation of surface area for MNS   **Mass of particle = (density) * (**$\frac{\boldsymbol{4}}{\boldsymbol{3}}$**π r^3^)** (density = 5.17 g/cm³ = 5170000 g/m^3^)  = 5170000 * $\frac{4}{3}$π $\frac{d^3}{8}$ (diameter/size of MNS)  **Mass of 1 MNS = 2705633.33 d^3^**  No. of particles per g = 1/(mass of 1 particle), surface area of particle = 4πr^2^  **Surface area (m^2^ /g) = (No. of MNS per g) *(surface area of the MNS)**  Surface area (m^2^ /g) = 3.14* d^2^/2705633.33 d^3^ (3.14/487014000)  MNS size (diameter) = 180 nm  Surface area (m^2^ /g) = 1160.54/180  = 6.44 m^2^ /g | |

**Table S2**.  Loading characteristics of MNP-APTES for NTD.

| Drug | Batches | Weight % ratio  (MNS-APTES: NTD) | Loading capacity  (%) | Loading efficiency (%) |
| --- | --- | --- | --- | --- |
| Nintedanib | A | 1:0.5 | 2.88 | 57.55 |
|  | B | 1:1 | 6.46 | 64.64 |
|  | C | 1:2 | 14.11 | 70.54 |
|  | D | 1:3 | 23.64 | 78.79 |
|  | E | 1.4 | 22.53 | 66.42 |
|  | F | 1:5 | 21.71 | 43.43 |

**Docking analysis of FGFR-4 Nintedanib complex**

Molecular docking analysis is a process to predict the best binding poses of protein and ligand interactions. The docking approach allows the understanding of binding interactions of small molecules at the binding site of target proteins to regulate fundamental biochemical processes. The prediction of various types of interactions is measured by calculating interaction energies and bond distances between each amino acid residue and ligand in the proximity of the pocket of the receptor. The binding of NTD in an active site of FGFR-4 kinase domain was represented in the surface image, as displayed in **Fig. S3(a)**. Additionally, the NTD poses with their pharmacophoric features considered for ligand interaction are highlighted in **Fig. S3 (b) and (c)**. After ten runs, the best docking poses with good interactions at the active site of the protein were calculated based on the interacting residues, type of interactions, lowest binding energy, and dock score (**Table S3)** within a proximity of 4.5 Å. The overlay of NTD with the native ligand at the active site in the binding pocket (kinase domain) of the protein showed that the confirmation and binding mode of the best-docked pose. These features are consistent with the ponatinib and have piperazine nitrogen for inhibitory action **Fig. S4(a)**.


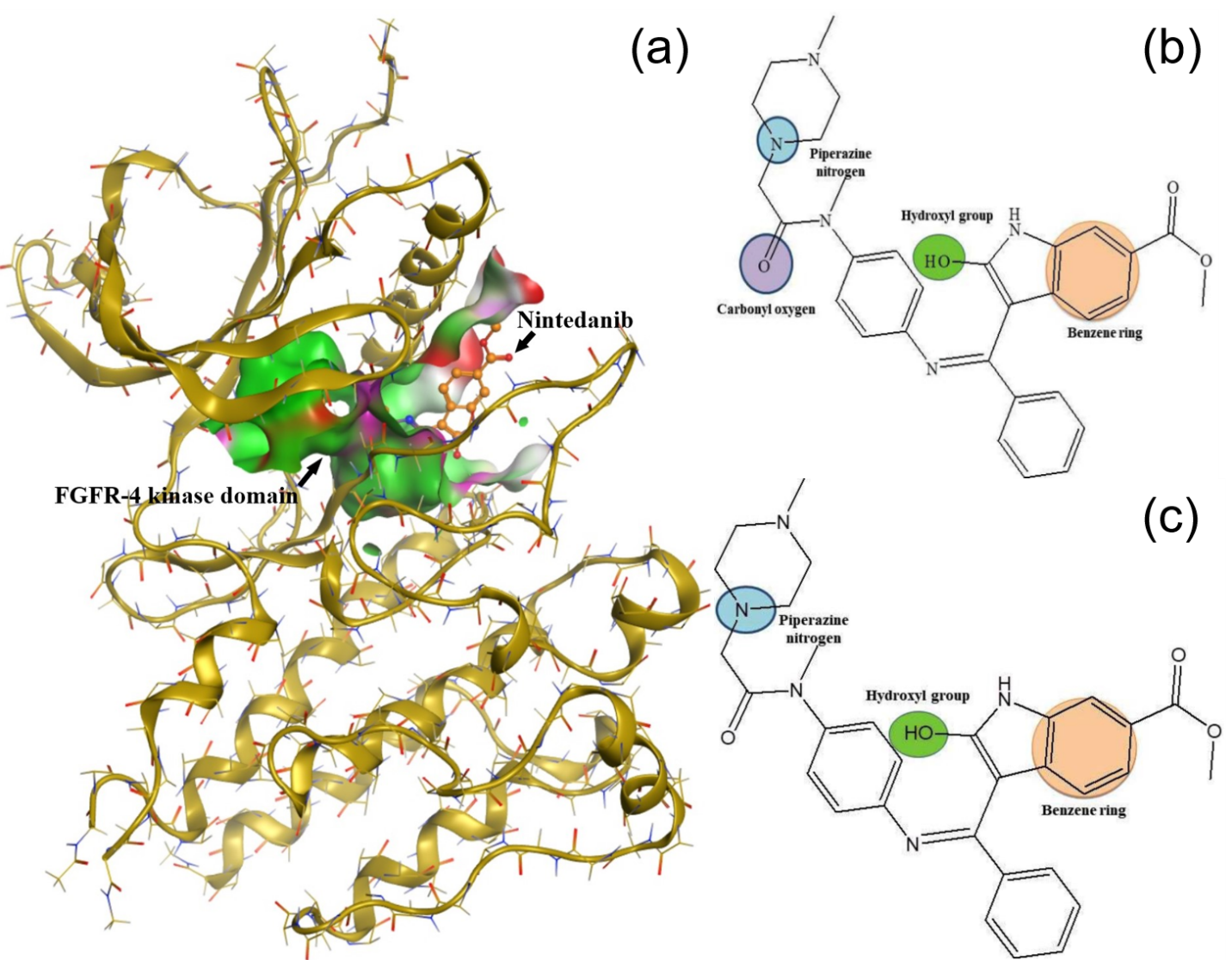


**Figure S3.** a) Surface representation of the docked complex of FGFR-4 and NTD b) and c) Chemical structure and pharmacophore of NTD in pose 3 and pose 7, respectively.

The docking results showed Ala554, Arg616, Asn648, Leu473, Met524, Ile609, Val523, His610, and Asp630 represent key residues in the active site of the pocket of the docked complex and contribute H-donor, H-acceptor, pi-H and ionic interactions with ligands. The best binding poses, *i.e.*, NTD -Pose-3 and NTD -Pose-7, showed lowest binding free energies as -4.9 and -1.7 kcal/mol in comparison to the native ligand -17.8 kcal/mol due to hydrogen bond formation and hydrophobic interactions. The kinase domain residues comprise Ala554, Arg616, Asn648, Met524, and Ile609 are involved in hydrogen bonding with piperazine nitrogen, carbonyl oxygen, and the hydroxyl group of NTD, whereas Leu473, Val523 are involved in hydrophobic π-π stacking interactions with the benzene ring as shown in **Fig. S4 (b) and (c)**. NTD-Pose-7 and native ligand Ponatinib showed hydrogen bond formation (H-donor) at Met524 residue with a distance of 3.29 Å and 2.71 Å, respectively. All the interactions have a significant role in stabilizing the confirmations of the protein-ligand complex. The amino acid residue Met524 is involved in the binding of NTD with the protein, which is the best suitable site for inhibition of FGFR-4.

**Table S3.** Ligand interactions of the docked complex of FGFR-4 (PDB id: 4UXQ)

| Receptor | Ligand pose | Nin----amino acid residues interaction | Interaction type | Distance,  Å | Total binding free energy, kcal/mol | RMSD |
| --- | --- | --- | --- | --- | --- | --- |
| FGFR-4  (4UXQ) | Nintedanib-Pose-3 | Nin-O72----Ala554 | H-donor | 2.99 | -4.9 | 3.088 |
|  |  | Nin-N12----Arg616 | H-acceptor | 3.59 |  |  |
|  |  | Nin-O23----Asn648 | H-acceptor | 3.13 |  |  |
|  |  | Nin-6-ring----Leu473 | pi-H | 4.27 |  |  |
| FGFR-4  (4UXQ) | Nintedanib-Pose-7 | Nin-N12----Met524 | H-donor | 3.29 | -1.7 | 2.506 |
|  |  | Nin-O72----Ile609 | H-donor | 3.05 |  |  |
|  |  | Nin-6-ring----Val523 | pi-H | 4.05 |  |  |
| FGFR-4  (4UXQ) | Ponatininb | Pon-N2----Met524 | H-donor | 2.71 | -17.8 | 2.359 |
|  |  | Pon-N4---- His610 | H-donor | 2.93 |  |  |
|  |  | Pon-O1---- Asp630 | H-acceptor | 3.10 |  |  |
|  |  | Pon-N4---- Asp630 | Ionic | 2.90 |  |  |

| 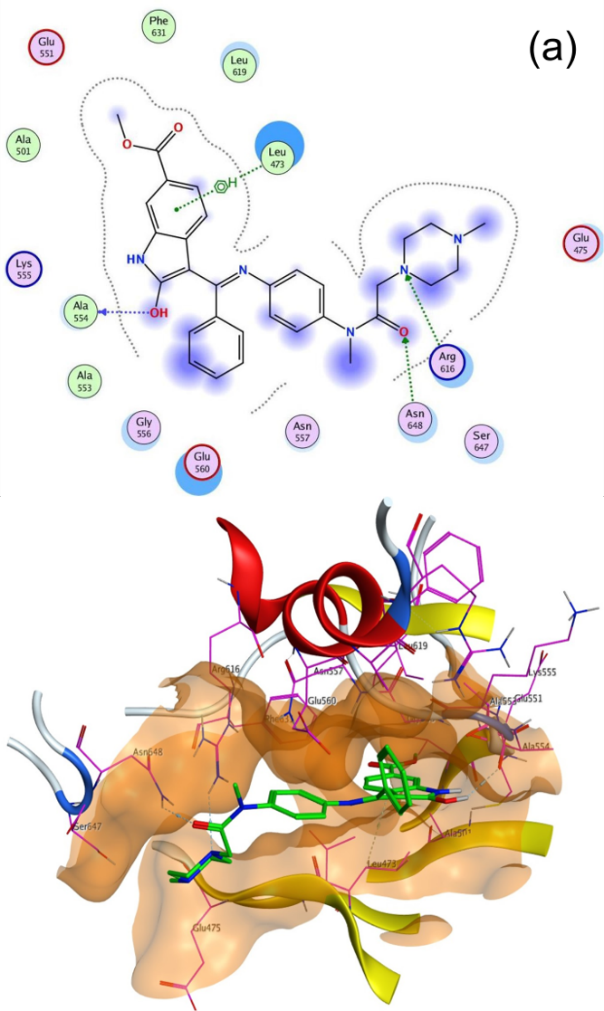 | 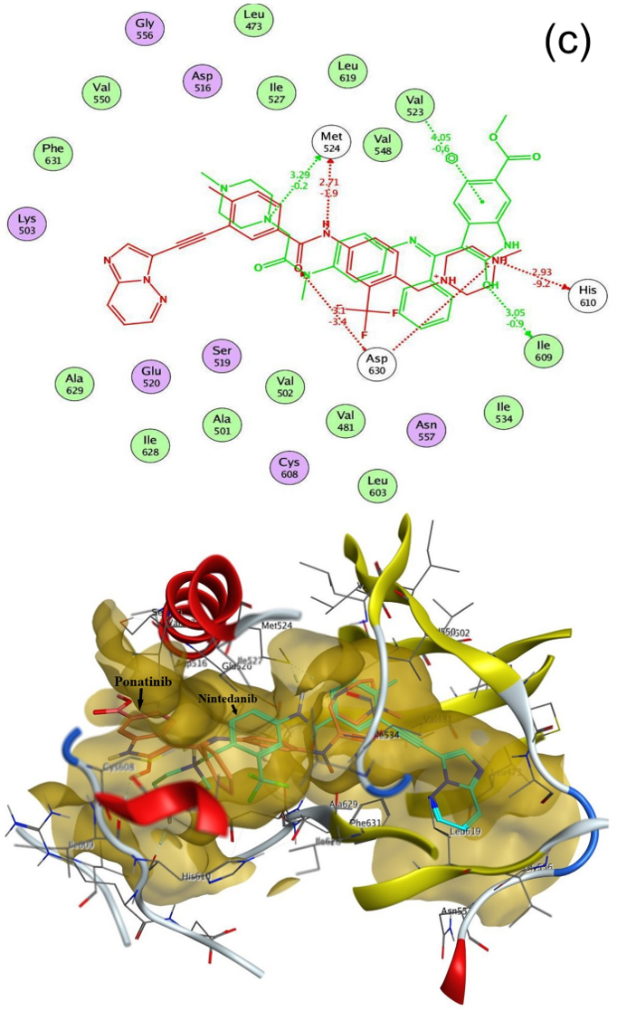 |
| --- | --- |
| 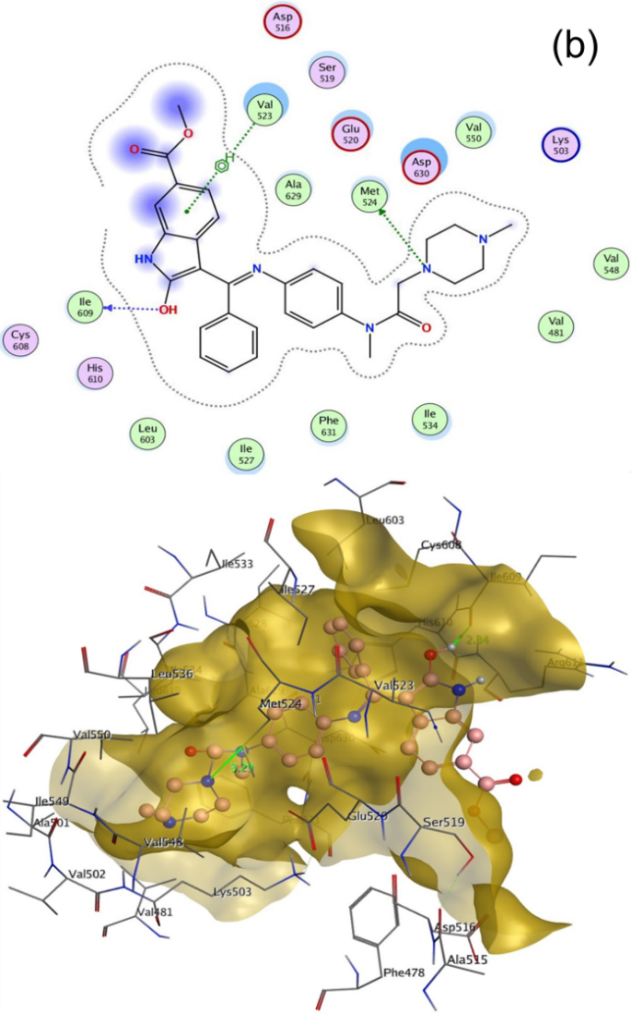 | **Figure S4.** Docked complex of FGFR-4 and NTD in 2D and 3D representation **a)** NTD (sticks form) and binding pose-3 b) NTD (ball & stick) and binding pose-7 with hydrogen bond and arene-π (pi-H) interactions at FGFR-4 (PDB id: 4UXQ) active site and c) Overlay of NTD (green) and Ponatinib (red) with binding energies and binding distance. |

**References**

S1. Molecular Operating Environment (MOE), 2015.08; Chemical Computing Group Inc.: Montreal, QC, Canada, 2015.

S2. Tucker et al., Structural Insights into FGFR Kinase Isoform Selectivity: Diverse Binding Modes of AZD4547 and Ponatinib in Complex with FGFR1 and FGFR4, (2014), Structure, 22, 1–11. http://dx.doi.org/10.1016/j.str.2014.09.019.
